# Effect of Coronary Computed Tomography Angiography Disease Burden on the Incidence of Recurrent Chest Pain

**DOI:** 10.1155/2014/304825

**Published:** 2014-07-02

**Authors:** Homayoun R. Ahmadian, Dustin M. Thomas, David J. Shaw, Megan L. Barnwell, Ronald L. Jones, Ryan J. McDonough, Ryan L. Prentice, Charles K. Lin, Ahmad M. Slim

**Affiliations:** Cardiology Service MCHE-MDC, Brooke Army Medical Center, 3551 Roger Brooke Drive, San Antonio, TX 78234-6200, USA

## Abstract

*Introduction.* The purpose of this study is to investigate chest pain evaluations after initial coronary computed tomography angiography (CCTA) based upon coronary artery disease (CAD) burden. *Methods.* CCTA results of 1,518 patients were grouped based on the CCTA results into no CAD, nonobstructive CAD (<50% maximal diameter stenosis), or obstructive CAD (≥50% stenosis). Chest pain evaluation after initial CCTA and rates of major adverse cardiovascular events (MACE) defined as the incidence of all-cause mortality, nonfatal MI, ischemic stroke, and late revascularization (>90 days following CCTA) were evaluated. *Results.* MACE rates were higher with obstructive CAD compared to nonobstructive CAD and no CAD (8.9% versus 0.7%, *P* < 0.001; 8.9 versus 1.6%, *P* < 0.001). One hundred seventy-four patients (11.5%) underwent evaluation for chest pain after index CCTA with rates significantly higher with obstructive CAD compared to both nonobstructive CAD and no CAD (7.5% versus 13.9% versus 17.8%, *P* < 0.001). The incidence of repeat testing was more frequent in patients with obstructive CAD (no CAD 36.5% versus nonobstructive CAD 54.9% versus obstructive CAD 67.7%, *P* = 0.015). *Conclusion.* Absence of obstructive disease on CCTA is associated with lower rates of subsequent evaluations for chest pain and repeat testing with low MACE event rates over a 22-month followup.

## 1. Introduction

Coronary CT angiography (CCTA) has emerged as an effective tool for both outpatient and emergency department (ED) evaluation of low to intermediate risk patients presenting with acute chest pain syndromes [1–6]. CCTA has also been shown effective as a complementary test in patients with equivocal or abnormal noninvasive results without high risk features [7]. The high negative predictive value (~99%) associated with maximal lumen diameter stenosis values <50% allows for safe disposition and potentially avoids the cost and risk associated with invasive coronary angiography (ICA) [8]. This has resulted in guideline statements recommending CCTA for these indications [9]. Recent updates to hospital inpatient reimbursement emphasize minimizing short stay inpatient hospitalizations under which chest pain rule-outs frequently fall [10]. This places a premium on the safe and efficient evaluation of this common symptom presentation that is cost effective.

Recurrent chest pain presentations often lead to repeat testing and recurrent hospitalizations. Recurrent emergency department evaluations and hospitalizations for chest pain have been reported previously without commenting on CCTA disease burden [11, 12]. We attempt to investigate recurrent chest pain evaluation after initial evaluation with coronary computed tomography angiography (CCTA) between those with previous obstructive CAD versus those with nonobstructive or no CAD as well as a comparison for major cardiac adverse events (MACE) in these groups.

## 2. Methods

Coronary CT angiography (CCTA) results from January 2005 until July 2012 at a high volume, single center tertiary referral hospital were retrospectively reviewed. A study population of 1518 patients evaluated in emergency department (ED), inpatient, or outpatient setting for the indications of chest pain, dyspnea, syncope, evaluation of graft/stent patency, or possible anomalous coronary artery was identified. Patients were grouped based on the CCTA results demonstrating either no CAD, nonobstructive CAD (<50% maximal diameter stenosis), or obstructive CAD (≥50% stenosis). Recurrent chest pain was determined based on ICD-9 codes. Rates of major adverse cardiovascular events (MACE) were abstracted.

CCTA images were analyzed by a cardiologist with level III ACC/ACR certified imaging expert in accordance with SCCT guidelines [13]. From January 2005 to December 2007, images were obtained using a 16-slice CT scanner (Brilliance-16R, Phillips, Amsterdam, Netherlands). From January 2008 to March 2011, images were obtained using a retrospective helical protocol with a 64 slice CT scanner (Somatom Definition CTR, Siemens, Erlagen, Germany). From March 2011 to March 2012, images were obtained utilizing a prospective sequential protocol with 60–80% image acquisition window. In March 2012 to July 2012, images were obtained using a 128-slice dual head scanner with a single heart beat image acquisition of the complete coronary when a heart rate of less than 60 was achieved (Somatom Definition Flash CTR, Siemens, Erlagen, Germany).

The primary endpoint was recurrent chest pain evaluation. Recurrent chest pain evaluations were identified initially by searching ICD-9 codes for atypical chest pain (786.59), chest pain (786.5), and angina (413) both in the inpatient and outpatient setting. All identified chest pain diagnoses after initial CCTA were manually adjudicated by per patient chart review. Any additional invasive or noninvasive cardiac risk stratification testing performed after the initial CCTA was documented, including multiple testing when applicable. Centers for Medicare and Medicaid Services (CMS) reimbursement rates, released in December 2013, were used to calculate per patient cost for additional risk stratification testing following CCTA. The composite secondary outcome was MACE, defined as all-cause mortality, stroke, nonfatal MI, and late revascularization, defined as revascularization performed within 90 days of CCTA with CAC imaging. ICD-9 codes for all-cause mortality (798.1, 798.2, 798.9, and V12.53), stroke (434.00, 434.01, 434.10, 434.11, 434.90, 434.91, 997.02, and V12.54), nonfatal MI (410.0–410.9), and late revascularization with PCI (92980, 92981, 92982, 92995, and 92996) or CABG (33510–33514, 33516, and 33533–33536) were used for initial data extraction followed by Department of Defense (DOD) outpatient and inpatient electronic medical records (EMR) verification of events. We determined mortality using the social security death index (SSDI) followed by reverification using EMR for last visit date as well as Tricare Healthcare Informatics Division verification. All events identified by ICD-9 code were adjudicated.

Statistical analysis was performed using IBM SPSS version 19.0 (IBM, Arnock, New York). Continuous variables are presented as means ± standard deviation. Categorical variables are presented as frequencies with percentages. Comparison of means between the various CCTA groups was performed using one-way ANOVA with *P* values <0.05 considered statistically significant. Kaplan-Meier method was used to demonstrate chest pain-free survival in the recurrent chest pain population as well as total survival free from MACE in the total population with a log rank value <0.05 considered statistically significant.

## 3. Results

One thousand five hundred eighteen patients underwent CCTA in an ED, inpatient or outpatient setting.  Table 1 shows the baseline demographic data and outcomes for the total patient cohort. Gender was equally distributed amongst the groups; however, the mean age increased with increasing CCTA disease burden (*P* < 0.001). CCTA evaluation in these 1518 patients at index evaluation demonstrated obstructive CAD, nonobstructive CAD, and no CAD in 11.5%, 43.1%, and 45.4%, respectively. Patients with obstructive CAD on CCTA were more likely to have documented diabetes mellitus (*P* < 0.001) and hypertension (*P* < 0.001). Obstructive CAD and nonobstructive CAD patients were more likely to have hyperlipidemia than no CAD CCTA patients (*P* < 0.001). Of the 174 patients with obstructive disease, single-vessel, 2-vessel, and 3-vessel obstruction was found in 64.5%, 32.3%, and 3.2%, respectively. All-cause mortality was rare in this patient population with 3 total deaths and was not statistically different stratified by CAD burden. Stroke, nonfatal MI, late revascularization, and composite MACE rates were significantly higher in the obstructive CAD cohort (*P* values <0.001, 0.003, <0.001, and <0.001, resp.) with overall low event rates.

**Figure 1:**
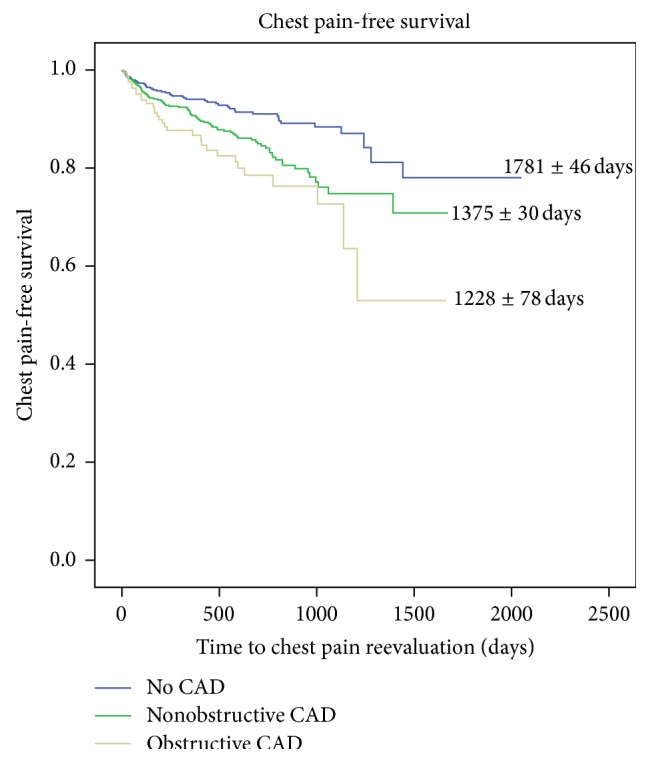
Kaplan-Meier curve demonstrating survival free from chest pain reevaluation based on CCTA disease burden.

**Figure 2:**
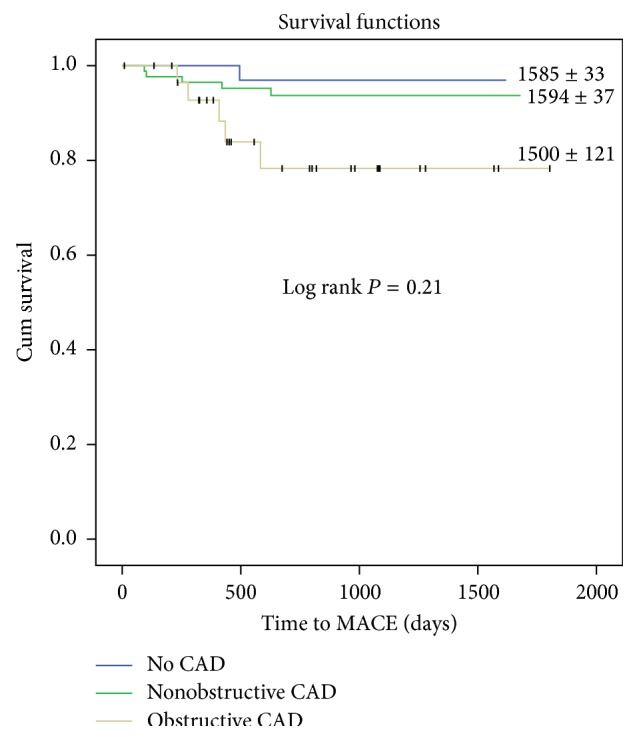
Kaplan-Meier curve demonstrating MACE free survival within the chest pain reevaluation population based on CCTA disease burden.

Recurrent chest pain evaluation occurred in 174 patients (11.5%) overall. Fifty-two patients with no CAD, 91 patients with nonobstructive CAD, and 31 patients with obstructive CAD (Table 2) presented for recurrent chest pain evaluation (7.5% versus 13.9% versus 17.8%, *P* < 0.001). The mean age at the time of reevaluation differed amongst the groups; however the nonobstructive CAD patients tended to be older at time of reevaluation (mean age 58.8 years, *P* = 0.029). The incidence of repeat testing (Table 2) was more frequent in patients with obstructive CAD (no CAD 36.5% versus nonobstructive CAD 54.9% versus obstructive CAD 67.7%, *P* = 0.015). This correlated with increased frequency of hospitalizations at initial reevaluation amongst the groups with 34.6%, 53.8%, and 58.1% of patients in the no CAD, nonobstructive CAD, and obstructive CAD groups, respectively (*P* = 0.046). There was no difference observed with respect to the mean duration of time between index CCTA and reevaluation for chest pain (*P* = 0.938) nor the length of stay (LOS) during hospitalization at first reevaluation (*P* = 0.810). At the time of reevaluation, review of initial CCTA results was significantly correlated with time free of recurrent chest pain with those having no CAD having a longer chest pain-free survival period with log rank showing *P* < 0.001 (Figure 1). Health care reevaluations for chest pain, including second, third, and fourth additional evaluations, were evaluated and were found not to be statistically significant amongst the three CCTA disease groups. Overall MACE free survival in the chest pain reevaluation cohort was significant for more events in the obstructive CAD group when compared to nonobstructive CAD and no CAD (log rank = 0.021) (Figure 2).

Outcomes and ability to predict recurrent chest pain evaluations and MACE were broken down into individual major epicardial coronary vessels and analyzed with respect to CAD burden. Obstructive LM CAD (Table 3) on initial CCTA was associated with higher rates of hospitalization at reevaluation (*P* = 0.032), high per patient cost in repeat testing (*P* = 0.019), higher all-cause mortality (*P* < 0.001), and composite MACE rates (*P* < 0.001). There was no difference between nonobstructive LM CAD, obstructive LM CAD, and no LM CAD with respect to chest pain reevaluations (13.5% versus 14.3% versus 11.1%, resp.  *P* = 0.563). Nonobstructive LM CAD was associated with longer hospital LOS (1.8 ± 1.3 days) when compared with no LM CAD and obstructive LM CAD (1.2 ± 0.5 days versus 1.0 ± 0.0 days, *P* = 0.005).

Obstructive LAD CAD (Table 4) was associated with higher rates of chest pain reevaluation (*P* < 0.001), hospitalization at reevaluation (*P* < 0.001), repeat testing at the time of reevaluation (*P* < 0.001), average per patient testing and cost (*P* < 0.001 for both), rates of myocardial infarction (*P* = 0.006), late revascularization (*P* = 0.006), and composite MACE (*P* = 0.005).

Obstructive LCX CAD (Table 5) was found to be associated with higher rates of chest pain reevaluation (*P* = 0.048), hospitalization at reevaluation (*P* = 0.039), repeat testing at the time of reevaluation (*P* = 0.003), average per patient testing (*P* = 0.001), average per patient cost for repeat testing (*P* < 0.001), all-cause mortality (*P* = 0.027), stroke (*P* = 0.003), late revascularization (*P* = 0.020), and composite MACE (*P* < 0.001). Any degree of CAD in the LCX was associated with higher rates of MI than no LCX disease (*P* = 0.001).

Nonobstructive CAD in the RCA (Table 6) was associated with higher rates of chest pain reevaluation compared with obstructive RCA CAD and no RCA CAD (15% versus 12.3% versus 9.8%, resp. *P* = 0.016). Nonobstructive RCA CAD was also associated with higher rates of repeat testing (*P* < 0.001), average tests performed per patient (*P* = 0.001), and per patient testing cost (*P* < 0.001). Obstructive RCA CAD was associated with higher rates of hospitalization at time of reevaluation (*P* = 0.014), hospital LOS (*P* = 0.018), stroke (*P* < 0.001), MI (*P* = 0.001), late revascularization (*P* < 0.001), and composite MACE (*P* < 0.001).

One hundred seventy-four patients with obstructive CAD on CCTA were further broken down into 1-vessel disease, 2-vessel disease, or 3-vessel disease. Obstructive LM disease was grouped under 2-vessel disease for the purposes of this analysis.  Table 7 outlines the demographics and outcomes in this subgroup. There was an age difference observed between the groups with older patients tending to have more diffuse disease (*P* < 0.001). Male predominance was observed in patients with 3-vessel disease when compared to the other two groups (*P* = 0.041). Reevaluations for chest pain were found more commonly in patients with 2-vessel CAD when compared to 1-vessel and 3-vessel (22.7% versus 17.7% and 4.8%, *P* = 0.009). This increased incidence of reevaluation did not translate into a difference with regard to frequency of hospitalization, hospital LOS, frequency of repeat ischemic testing, or total number of repeat tests ordered per group. Additionally, the time to reevaluation and the incidence of morbidity and mortality were not different between the three groups. Within the obstructive disease cohort, twenty-one patients underwent repeat testing at the time of reevaluation when compared with 10 patients where testing was deferred (67.7% versus 32.3%). No difference was observed with regard to stroke (9.5% versus 0%, *P* = 0.313), MI (9.5% versus 10%, *P* = 0.967), late revascularization (19% versus 0%, *P* = 0.277), and composite MACE (33.3% versus 10%, *P* = 0.222) whether additional testing was performed or not (Table 8). There were no mortalities observed in the obstructive CAD cohort.

Testing frequency also appeared to be affected by CCTA disease burden. When comparing all patients with reevaluation for chest pain, the average number of total tests ordered was higher in the patients with obstructive disease (0.8 ± 0.7) when compared to patient with nonobstructive CAD or no CAD (0.7 ± 0.7 versus 0.4 ± 0.5, *P* = 0.006). Table 2 outlines approximate cost per test ordered. This resulted in a higher per patient cost in the obstructive CAD population when compared to nonobstructive CAD and no CAD patients ($255 versus $226 versus $98, *P* = 0.001). However, once patients were selected to undergo additional testing, there was no difference amongst the groups with regard to the average number of tests ordered per patient in the obstructive, nonobstructive, and no CAD groups (1.2 ± 0.5 versus 1.2 ± 0.4 versus 1.1 ± 0.2, *P* = 0.380). Hospital LOS was not different between the groups and thus was not included in the cost analysis. Outcomes were not different in the 90 patients that underwent repeat testing at time of reevaluation for chest pain when compared to the 84 patients where testing was deferred (Table 9). No deaths were observed and the rates stroke (2.2% versus 1.2%, *P* = 1.0), MI (2.2% versus 3.6, *P* = 0.673), late revascularization (4.4% versus 0%, *P* = 0.122), and composite MACE (7.8% versus 4.8%, *P* = 0.538) were not different in the repeat testing and no repeat testing groups, respectively.

## 4. Discussion

This retrospective analysis illustrates that a previous CCTA demonstrating the absence of CAD or nonobstructive disease is associated with a reduction in both emergency department and outpatient reevaluations for chest pain in addition to MACE. In addition to improved outcomes, we demonstrated a reduction in additional testing and per patient cost based on overall CCTA disease burden and individual epicardial coronary vessel CAD burden and stratified by 1-vessel, 2-vessel, and 3-vessel obstructive CAD. These findings further our understanding of both the prognostic utility and cost savings associated with the use of CCTA in acute and subacute chest pain evaluations.

Coronary artery disease is a major contributor of morbidity and mortality and effective means of evaluating those at increased risk of major cardiac events is paramount. At the same time, chest pain is a very common cause of emergency department (ED) presentations and return visits are common; therefore, an effective strategy to curtail these recurrent presentations is vital. CCTA has proven to be an effective means to evaluate patients with possible ACS in the emergency department who have low to intermediate risk of CAD [14]. Current 64-slice multidetector CTs have been shown to have a negative predictive value of approximately 99% [8]. From a prognostic standpoint, previous studies have shown that those who have the absence of CAD or nonobstructive disease on CCTA (defined as lesions causing less than 50% luminal stenosis) in the evaluation of acute chest pain have equally benign clinical outcomes during follow-up evaluations [11]. In opposition, the CONFIRM registry showed that those individuals with obstructive 2- or 3-vessel disease or obstructive left main disease had higher rates of all-cause mortality and nonfatal MI despite absence of symptoms [1].

Given the high costs associated with recurrent ED evaluations and inpatient admissions for chest pain, a more definitive strategy of excluding significant CAD was needed and CCTA seems to be providing this necessity. A recently published study comparing CCTA to standard evaluation in the emergency department triage of low-risk chest pain revealed that CCTA led to a statistically significant decrease in admission rates, and return visits to the ED for recurrent chest discomfort were 5 times more likely within 30 days in the standard evaluation arm [12]. The standard evaluation arm consisted of patients either discharged directly from ED after evaluation or inpatient admission ± stress testing.

Further studies have been done comparing CCTA to myocardial perfusion imaging in the evaluation of acute, low-risk chest pain patients in the ED. A recent multicenter randomized trial showed that compared to MPI, CCTA resulted in a statistically significant reduction in time to diagnosis and decreased health care costs without a difference in major adverse cardiac events after normal index findings [6].

CCTA not only allows prognostication based on presence or absence of obstructive disease but also allows for determination of plaque characterization that may reveal future presentations for chest pain due to acute coronary syndromes (ACS) [15]. These characteristics include “spotty” calcification, positive remodeling (defined as overall vessel diameter greater than 10% above reference vessel), and lower plaque attenuation less than 30 Hounsfield units [16].

Individual epicardial vessel CAD burden in this study demonstrated predictive utility for predicting repeat chest pain evaluations with the exception of LM obstructive disease. While this finding is unanticipated, it likely represents the very small number of patients (7 total patients) in the study cohort with obstructive LM CAD. Additionally, nearly all of these patients went on to coronary artery bypass graft (CABG) surgery within 30 days of initial CCTA. Thus with complete revascularization, lower recurrence rates for angina would be expected. An unpublished analysis by our group demonstrated the presence of LM coronary artery calcium (CAC) may predict MACE events to include stroke above that which would be predicted by total CAC score alone in a symptomatic cohort of patients. Indeed this current analysis also suggests the presence of obstructive LM disease predicts higher MACE rates to include mortality, stroke, MI, and late revascularization.

## 5. Conclusion

Absence of obstructive disease on CCTA is associated with much lower rates of subsequent evaluations for chest pain and repeat testing with associated low MACE event rates over 6-year review period and median followup of 22 months.

## Figures and Tables

**Table 1:** Baseline demographic data and chest reevaluation outcomes based on CCTA disease burden.

	No CAD *n* = 689 (%)	Nonobstructive CAD *n* = 655 (%)	Obstructive CAD *n* = 174 (%)	*P* value
Age, years	51.8 ± 13.9	60.9 ± 12.6	72.9 ± 17.7	<0.001
Male	410 (59.5)	391 (59.7)	118 (67.8)	0.113
Reevaluation for chest pain	52 (7.5)	91 (13.9)	31 (17.8)	<0.001
Diabetes mellitus	14 (2.0%)	31 (4.7%)	17 (9.8%)	<0.001
Hypertension	155 (22.5%)	307 (46.9%)	98 (56.3%)	<0.001
Hyperlipidemia	129 (18.7%)	266 (40.6)	72 (41.4%)	<0.001
All-cause mortality	2 (0.3%)	0 (0.0%)	1 (0.6%)	1.000
Stroke	3 (0.4%)	4 (0.6%)	6 (3.4%)	<0.001
Myocardial infarction	0 (0.0%)	6 (0.9%)	4 (2.3%)	0.003
Late revascularization	0 (0.0%)	1 (0.2%)	4 (2.3%)	<0.001
Composite MACE	5 (0.7%)	11 (1.7%)	14 (8.0%)	<0.001

**Table 2:** Reevaluations for chest pain based on CCTA disease burden.

	No CAD *n* = 52	Nonobstructive CAD *n* = 91	Obstructive CAD *n* = 31	*P* value
Age, years	52.8 ± 11.7	58.8 ± 13.5	56.4 ± 12.7	0.029
Male, *n* (%)	23 (44.2)	46 (50.5)	18 (58.1)	0.470
Repeat testing, *n* (%)	19 (36.5)	50 (54.9)	21 (67.7)	0.015
Repeat tests per patient	0.4 ± 0.5	0.7 ± 0.7	0.8 ± 0.7	0.006
Cost of additional testing per patient	$98	$226	$255	0.001
Mean time to 1st reevaluation, days	348 ± 375	355 ± 314	330 ± 332	0.938
Hospitalization at 1st reevaluation, *n* (%)	18 (34.6)	49 (53.8)	18 (58.1)	0.046
1st reevaluation mean LOS, days	1.2 ± 0.5	1.3 ± 0.7	1.6 ± 1.0	0.810
2nd reevaluation, *n* (%)	19 (36.5)	30 (33.0)	8 (25.8)	0.601
Mean time to 2nd reevaluation, days	567 ± 410	581 ± 301	439 ± 322	0.580
Hospitalizations at 2nd reevaluation, *n*	8 (15.4)	11 (12.1)	5 (16.1)	0.788
2nd reevaluation mean LOS, days	1.4 ± 0.7	1.6 ± 1.0	1.8 ± 1.8	0.810
3rd reevaluation, *n* (%)	4 (7.7)	8 (8.8)	3 (9.7)	0.949
Mean time to 3rd reevaluation, days	592 ± 451	658 ± 425	485 ± 461	0.843
Hospitalizations at 3rd reevaluation, *n*	0 (0)	0 (0)	0 (0)	n/a
3rd reevaluation mean LOS, days	n/a	1.0 ± 0.0	1.0 ± 0.0	1.000
4th reevaluation, *n*	0 (0)	3 (3.3)	1 (3.2)	0.418
Mean time to 4th reevaluation, days	n/a	1008 ± 354	180 ± 0	0.180
Hospitalizations at 4th reevaluation, *n*	0	2 (3.3)	0	0.249
4th reevaluation mean LOS, days	n/a	1.0 ± 0.0	n/a	n/a

**Table 3:** Baseline demographic data, outcomes, and cost analysis based on burden of CAD in the LM.

	No LM CAD *n* = 1289	Nonobstructive LM *n* = 222	Obstructive LM *n* = 7	*P* value
Age	51.8 ± 13.9	60.9 ± 12.6	72.9 ± 17.7	<0.001
Male gender	774 (60)	139 (62.6)	6 (85.7)	0.303
Chest pain reevaluation, *n* (%)	143 (11.1)	30 (13.5)	1 (14.3)	0.563
Time to reevaluation, days	353 ± 342	335 ± 304	100 ± 0	0.732
Hospitalization at reevaluation, *n* (%)	64 (5.0)	20 (9.0)	1 (14.3)	0.032
Hospital LOS, days	1.2 ± 0.5	1.8 ± 1.3	1.0 ± 0	0.005
Repeat testing, *n* (%)	70 (5.4)	19 (8.6)	1 (14.3)	0.122
Tests per patient, mean	0.06 ± 0.3	0.1 ± 0.4	0.1 ± 0.4	0.131
Cost per patient, mean	$18 ± 100	$37 ± 139	$83 ± 220	0.019
All-cause mortality, *n* (%)	3 (0.2)	0 (0)	1 (14.3)	<0.001
Stroke, *n* (%)	8 (0.6)	5 (2.3)	0 (0)	0.05
MI, *n* (%)	9 (0.7)	7 (3.2)	0 (0)	0.004
Late revascularization, *n* (%)	2 (0.2)	3 (1.4)	0 (0)	0.016
Composite MACE, *n* (%)	20 (1.6)	13 (5.9)	1 (14.3)	<0.001

LM: left main coronary artery.

**Table 4:** Baseline demographic data, outcomes, and cost analysis based on burden of CAD in the LAD.

	No LAD CAD *n* = 747	Nonobstructive LAD *n* = 631	Obstructive LAD *n* = 140	*P* value
Age	48.6 ± 14.1	57.4 ± 12.9	58.6 ± 12.2	<0.001
Male gender	448 (60)	380 (60.2)	91 (65)	0.524
Chest pain reevaluation, *n* (%)	63 (8.4)	85 (13.5)	26 (18.6)	<0.001
Time to reevaluation, days	358 ± 368	353 ± 308	309 ± 347	0.804
Hospitalization at reevaluation, *n* (%)	23 (3.1)	46 (7.3)	16 (11.4)	<0.001
Hospital LOS, days	1.2 ± 0.5	1.3 ± 0.8	1.5 ± 1.1	0.436
Repeat testing, *n* (%)	23 (3.1)	49 (7.8)	18 (12.9)	<0.001
Tests per patient, mean	0.03 ± 0.2	0.09 ± 0.3	0.16 ± 0.5	<0.001
Cost per patient, mean	$9 ± 65	$34 ± 134	$50 ± 168	<0.001
All-cause mortality, *n* (%)	3 (0.4)	0 (0)	1 (0.7)	0.193
Stroke, *n* (%)	3 (0.4)	7 (1.1)	3 (2.1)	0.081
MI, *n* (%)	3 (0.4)	11 (1.7)	2 (1.4)	0.047
Late revascularization, *n* (%)	0 (0)	3 (0.5)	2 (1.4)	0.006
Composite MACE, *n* (%)	9 (1.2)	17 (2.7)	8 (5.7)	0.005

LAD: left anterior descending.

**Table 5:** Baseline demographic data, outcomes, and cost analysis based on burden of CAD in the LCX.

	No LCX CAD *n* = 1029	Nonobstructive LCX *n* = 441	Obstructive LCX *n* = 48	*P* value
Age	51.3 ± 14.2	56.5 ± 13.2	62.9 ± 13.6	<0.001
Male gender	610 (59.3)	270 (61.2)	39 (81.3)	0.009
Chest pain reevaluation, *n* (%)	104 (10.1)	62 (14.1)	8 (16.7)	0.048
Time to reevaluation, days	358 ± 345	322 ± 311	441 ± 400	0.586
Hospitalization at reevaluation, *n* (%)	47 (4.6)	34 (7.7)	4 (8.3)	0.039
Hospital LOS, days	1.2 ± 0.7	1.4 ± 0.9	1.5 ± 0.6	0.382
Repeat testing, *n* (%)	49 (4.8)	34 (7.7)	7 (14.6)	0.003
Tests per patient, mean	0.05 ± 0.3	0.1 ± 0.4	0.2 ± 0.5	0.001
Cost per patient, mean	$15 ± 85	$35 ± 144	$75 ± 201	<0.001
All-cause mortality, *n* (%)	3 (0.3)	0 (0)	1 (2.1)	0.027
Stroke, *n* (%)	4 (0.4)	7 (1.6)	2 (4.2)	0.003
MI, *n* (%)	4 (0.4)	11 (2.5)	1 (2.1)	0.001
Late revascularization, *n* (%)	1 (0.1)	3 (0.7)	1 (2.1)	0.020
Composite MACE, *n* (%)	12 (1.2)	17 (3.9)	5 (10.4)	<0.001

LCX: left circumflex coronary artery.

**Table 6:** Baseline demographic data, outcomes, and cost analysis based on burden of CAD in the RCA.

	No RCA CAD *n* = 990	Nonobstructive RCA *n* = 454	Obstructive RCA *n* = 73	*P* value
Age	51.2 ± 14.1	56.6 ± 13.3	59.6 ± 14.0	<0.001
Male gender	599 (60.5)	268 (59)	51 (69.9)	0.213
Chest pain reevaluation, *n* (%)	97 (9.8)	68 (15)	9 (12.3)	0.016
Time to reevaluation, days	371 ± 349	326 ± 327	277 ± 215	0.567
Hospitalization at reevaluation, *n* (%)	43 (4.3)	36 (7.9)	6 (8.2)	0.014
Hospital LOS, days	1.2 ± 0.7	1.3 ± 0.6	2.2 ± 1.5	0.018
Repeat testing, *n* (%)	41 (4.1)	43 (9.5)	6 (8.2)	<0.001
Tests per patient, mean	0.05 ± 0.3	0.11 ± 0.4	0.08 ± 0.3	0.001
Cost per patient, mean	$14 ± 88	$40 ± 146	$34 ± 125	<0.001
All-cause mortality, *n* (%)	3 (0.3)	0 (0)	1 (1.4)	0.098
Stroke, *n* (%)	4 (0.4)	4 (0.9)	5 (6.8)	<0.001
MI, *n* (%)	4 (0.4)	9 (2.0)	3 (4.1)	0.001
Late revascularization, *n* (%)	0 (0)	3 (0.7)	2 (2.7)	<0.001
Composite MACE, *n* (%)	0 (0)	1 (0.2)	1 (1.4)	<0.001

RCA: right coronary artery.

**Table 7:** Outcomes in patients with obstructive CAD.

	1v CAD *n* = 113	2v CAD *n* = 44	3v CAD *n* = 21	*P* value
Age, years	56.7 ± 9.7	59.5 ± 14.3	66.1 ± 15.1	<0.001
Male, *n* (%)	76 (67.3)	27 (61.4)	18 (85.7)	0.041
Chest pain reevaluation, *n* (%)	20 (17.7)	10 (22.7)	1 (4.8)	0.009
Time to reevaluation, days	330 ± 326	345 ± 375	181 ± 0	0.955
Hospitalization at reevaluation, *n* (%)	11 (55)	6 (60)	1 (100)	0.665
Hospital LOS, days	1.4 ± 0.7	1.8 ± 0.7	2.0 ± 0.0	0.272
Repeat testing, *n* (%)	12 (60)	8 (80)	1 (100)	0.146
Total number of repeat tests, *n*	20	10	1	0.122
All-cause mortality, *n* (%)	0 (0)	0 (0)	0 (0)	n/a
Stroke, *n* (%)	2 (10)	0 (0)	0 (0)	0.555
MI, *n* (%)	2 (10)	1 (10)	0 (0)	0.946
Composite MACE, *n* (%)	4 (20)	1 (10)	0 (0)	0.283

1v: single-vessel Coronary artery disease (CAD); 2v: 2-vessel CAD; 3v: 3-vessel CAD; LOS: length of stay; MI: myocardial infarction; composite MACE: combined endpoint to all-cause mortality, stroke, MI, and late revascularization.

**Table 8:** Outcomes in all patients undergoing reevaluation for recurrent chest pain based on whether repeat testing was performed at any point during the follow-up period.

	Repeat testing *n* = 90 (%)	No repeat testing *n* = 84 (%)	*P* value
All-cause mortality	0 (0)	0 (0)	n/a
Stroke	2 (2.2)	1 (1.2)	1.0
MI	2 (2.2)	3 (3.6)	0.673
Late revascularization	4 (4.4)	0 (0)	0.122
Composite MACE	7 (7.8)	4 (4.8)	0.538

MI: myocardial infarction; composite MACE: combined endpoint to all-cause mortality, stroke, MI, and late revascularization.

**Table 9:** Obstructive CAD patient outcomes (1v, 2v, or 3v CAD) based on whether patients were retested at any point during the follow-up period.

	Repeat testing *n* = 21 (%)	No repeat testing *n* = 10 (%)	*P* value
All-cause mortality	0 (0)	0 (0)	n/a
Stroke	2 (9.5)	0 (0)	0.313
MI	2 (9.5)	1 (10)	0.967
Late revascularization	4 (19)	0 (0)	0.277
Composite MACE	7 (33.3)	1 (10)	0.222

MI: myocardial infarction; composite MACE: combined endpoint to all-cause mortality, stroke, MI, and late revascularization.
